# Development and characterization of a recombinant Bundibugyo virus expressing a fluorescent reporter protein

**DOI:** 10.1080/22221751.2026.2709847

**Published:** 2026-07-24

**Authors:** Shilpi Jain, Éric Bergeron, Mike Flint, Joel M. Montgomery, Christina F. Spiropoulou, César G. Albariño

**Affiliations:** Viral Special Pathogens Branch, Division of High Consequence Pathogens and Pathology, Centers for Disease Control and Prevention, Atlanta, GA, USA

**Keywords:** Bundibugyo virus, BDBV, Ebola virus, neutralization, antivirals, outbreak

## Abstract

Bundibugyo virus disease (BVD), caused by Bundibugyo virus (BDBV), is associated with substantial morbidity and mortality, with previous outbreaks reporting case fatality rates of 30%–50%. The ongoing BDBV outbreak in the Democratic Republic of the Congo and Uganda highlights the urgent need for virus-specific research tools and medical countermeasures. Unlike Ebola virus disease caused by Zaire ebolavirus, no licensed vaccines or specific therapeutics are currently available for BVD. The lack of research tools has limited studies with BDBV. To facilitate antiviral testing and neutralization studies, we developed the first recombinant BDBV expressing the fluorescent reporter protein ZsGreen (rBDBV-ZsG). As a proof of concept, we tested a set of previously characterized antiviral compounds and demonstrated comparable inhibitory profiles between rBDBV-ZsG and the wild-type BDBV parental strain. Furthermore, the utility of rBDBV-ZsG was successfully evaluated in neutralization assays, demonstrating robust sensitivity and specificity using monoclonal antibodies and convalescent serum samples.

## Introduction

The Bundibugyo virus (BDBV; *Orthoebolavirus bundibugyoense*) was first identified in 2007 during an outbreak in Uganda [1]. A subsequent BDBV outbreak occurred in the Democratic Republic of the Congo (DRC) in 2012 [2]. In these outbreaks, the reported case fatality rate (CFR) for BDBV has ranged from 30% to 50% [3]. In May 2026, an outbreak of Ebola disease caused by BDBV emerged in the DRC and Uganda and rapidly escalated to a WHO-declared Public Health Emergency of International Concern [4]. Unlike Ebola virus disease caused by Zaire ebolavirus (ZEBOV), there is currently no licensed vaccine or specific therapeutic available for Bundibugyo virus disease (BVD) [5]. Despite urgent international efforts, the basic research tools needed to respond effectively to this outbreak remain limited. Previously, we and others developed various filoviruses expressing the reporter protein [6–12]. However, no published reverse genetics system currently exists for BDBV, severely limiting mechanistic studies and therapeutic development.

Here, we describe the first recombinant BDBV expressing the fluorescent reporter protein ZsGreen (rBDBV-ZsG), based on the BDBV 2012 strain. As proof of concept, we first tested a panel of previously described antiviral compounds effective for ZEBOV in our BSL-4 laboratories and evaluated their specificity using both rBDBV-ZsG and wild-type BDBV 2012 strain. We further assessed the utility of rBDBV-ZsG for neutralization studies.

## Results

### Design and rescue of a reporter BDBV

**Figure 1.**
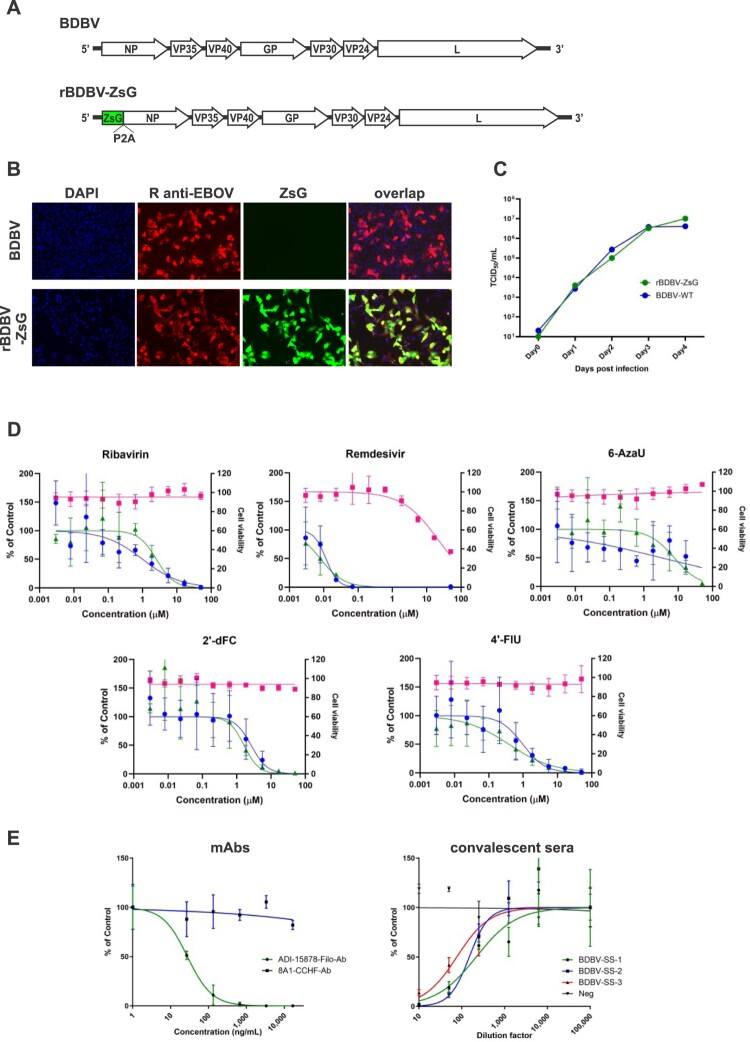
(A) Schematic representation of the genomes of recombinant Bundibugyo virus (rBDBV) with or without ZsGreen (ZsG) protein. (B) Immunofluorescence assay (IFA) showing Vero-CCL-81 cells infected with wild-type BDBV (BDBV) or rBDBV-ZsG (magnification 20 X; EVOS microscope). (C) Growth curves of indicated viruses in Huh7 cells (Multiplicity of Infection: 0.1). (D) Concentration-response curves in Huh7 cells infected with BDBV-WT (blue circles) or rBDBV-ZsG (green triangles) after treatment with various antiviral compounds. (E) Left panel: Concentration-response curves in Vero-CCL-81 cells infected with rBDBV-ZsG after neutralization with anti-filovirus (ADI-15878; green line) and anti-CCHFV (8A1; blue line) antibodies. Right panel: Concentration-response curves in Vero-CCL-81 cells infected with rBDBV-ZsG after neutralization with the archived human serum samples.

The full-length BDBV clone was generated and rBDBV-ZsG was successfully rescued as described in Materials and Methods (Figure 1A and Supp Figure 1). Since the functional compatibility of EBOV and BDBV polymerase complex has been well-demonstrated [13], we used our previously developed EBOV support plasmids to rescue rBDBV-ZsG. Virus rescue was confirmed by IFA using a polyclonal rabbit anti-EBOV antibody (Figure 1B) and by next-generation sequencing (Illumina MiniSeq System). rBDBV-ZsG exhibited growth kinetics similar to those of the corresponding wild-type virus (Figure 1C).

### Testing of rBDBV-ZsG in the presence of antiviral compounds

To evaluate antiviral compounds, we tested a panel of well-characterized compounds with both rBDBV-ZsG and BDBV-WT. The viruses were used to compare antiviral activity in the presence or absence of the reporter protein. To assess the suitability of rBDBV-ZsG for antiviral screening, we used 50 µM ribavirin as the positive control and DMSO vehicle-only treatment as the negative control. The Z’ score for rBDBV-ZsG was calculated to be 0.57, indicating that the reporter virus is suitable for antiviral compound screening. The signal-to-noise ratios for rBDBV-ZsG and BDBV-WT were 254:1 and 34:1, respectively.

A concentration-dependent reduction was observed in cells infected with rBDBV-ZsG across a range of ribavirin concentrations, and a comparable reduction in infected cells was observed with BDBV-WT. The EC₅₀ values for rBDBV-ZsG and BDBV-WT were 2.5 and 1 µM, respectively (Supp Figure 3A), with no loss of cell viability at the concentrations tested. These values are similar to those previously reported for Zaire ebolavirus [7,8]. We next tested additional viral polymerase inhibitors, including remdesivir, 2′-deoxy-2′-fluorocytidine (2’-dFC), 6-azauridine (6-AzaU), and 4′-fluorouridine (4′-FlU; EIDD-2749). All compounds tested produced dose-dependent inhibition curves (Figure 1D).

### Pan-filovirus monoclonal antibody efficiently neutralizes rBDBV-ZsG

We tested rBDBV-ZsG in a neutralization assay using the well-characterized monoclonal antibody ADI-15878, which targets multiple ebolavirus species [14]. ADI-15878 efficiently neutralized rBDBV-ZsG, whereas no neutralizing activity was detected with the control antibody targeting Crimean-Congo hemorrhagic fever virus (CCHFV). In addition, quantification of ZsGreen expression showed that ADI-15878 neutralized rBDBV-ZsG in a dose-dependent manner, with an IC_50_ value of 28 ng/mL (Figure 1E). This IC_50_ is comparable to the value reported previously [14] for BDBV, confirming the utility of the reporter virus for doing neutralization studies.

### Neutralization assays using archived clinical sera

After confirming the utility of rBDBV-ZsG for neutralization studies, we tested archived human serum samples collected during the 2007 BDBV outbreak in Uganda for neutralizing activity against rBDBV-ZsG. All archived human serum samples tested exhibited neutralizing activity against rBDBV-ZsG, whereas no neutralizing activity was observed with the control serum sample (Figure 1E).

## Discussion

The ongoing BDBV outbreak in the DRC and Uganda represents the largest recorded outbreak caused by BDBV. The public health response is particularly challenging because no vaccines or therapeutics are currently approved specifically for BDBV [15]. In addition, the limited availability of experimental tools for studying BDBV has hindered efforts to characterize viral replication, evaluate antibody-mediated neutralization, and screen antiviral compounds. In this study, we developed a recombinant BDBV reverse-genetics system based on the 2012 BDBV strain. Because the 2012 strain is closely related to the currently circulating BDBV strain (Supp Figure 2), we used this backbone to generate a recombinant reporter virus expressing the fluorescent protein ZsGreen.

Although our previously developed support plasmids for EBOV allowed a highly efficient rescue of the homologous virus, we observed a lower rescue efficiency for BDBV. In our next studies, we are planning to develop homologous support plasmids to potentially improve the efficiency of our BDBV reverse genetics system.

Here, we demonstrate that viral polymerase inhibitors previously reported to be effective against ZEBOV are also active against BDBV. In this study, we confirmed the potent antiviral activity of remdesivir against BDBV, with an EC₅₀ of approximately 10 nM as previously reported [7].

Our study further shows that archived serum samples from 2007 BDBV outbreak neutralized BDBV-ZsG efficiently, suggesting that these individuals have BDBV-specific neutralizing antibody activity. Due to the high similarity between all BDBV strains, we anticipate that these samples neutralize even 2026 BDBV strain. However, this possibility requires further evaluation using a larger number of samples and, ideally, neutralization assays performed directly against the 2026 strain. Overall, these findings suggest that cross-neutralization may occur among different BDBV strains. Additional studies are needed to define the breadth and durability of BDBV-specific neutralizing antibody responses.

In conclusion, we describe the development of a recombinant BDBV reporter virus expressing ZsG. It is a useful tool for studying BDBV replication and pathogenesis. The findings from this study may help inform the development and evaluation of medical countermeasures for the ongoing BDBV outbreak.

## Institutional review board statement

This research was approved by CDC Institutional Review Board (IRB Protocol #7276).

## Disclaimer

The findings and conclusions in this report are those of the authors and do not necessarily represent the official position of the CDC. The mention of company names or products does not constitute endorsement by CDC.

## Supplementary Material

SuppFig2 GP alignment.pdf

SuppFig1 cloning strategy.pdf

SuppFig3 tables.pdf

Materials and Methods_revision1_clean.docx

## Use of generative artificial intelligence

Generative artificial intelligence (AI) tools were used to support language refinement and clarity in manuscript preparation. Specifically, ChatGPT (OpenAI; GPT-5) was used to assist with improving grammar, structure, and readability of text drafted by the authors. These tools were not used for data analysis, interpretation of results, generation or manipulation of figures, creation of original research data, or development of scientific conclusions.

All outputs were critically reviewed, edited, and verified by the authors, who take full responsibility for the originality, accuracy, and integrity of the content.
