# Supplementary material for: Development and characterization of a recombinant Bundibugyo virus expressing a fluorescent reporter protein
Source: Emerg Microbes Infect. 2026 Jul 24;15(1):2709847. doi: 10.1080/22221751.2026.2709847 (PMC13435268; doi:10.1080/22221751.2026.2709847)
Supplement: Supplementary file 1 [file TEMI_A_2709847_SM3041.pdf]

Supp.Fig.3

A

	EC_{50} (μM)			SI	
	BDBV-2012-WT	rBDBV-2012-ZsG	CC_{50} (μM)	BDBV-2012-WT	rBDBV-2012-ZsG
**Ribavirin**	1	2.5	>50	>50	>20
**Remdesivir**	0.012	0.008	21	1750	2625
**6-AzaU**	3.3	7.7	>50	>15	>7
**2'dFC**	2.6	1.6	>50	>19	>31
**4'-FlU**	0.98	0.39	>50	>51	>128

B

mAbs	IC_{50} (ng/ml)
ADI-15878-Filo-Ab	28
8A1-CCHF-Ab	$>10^5$

Conv. sera	Neut. Titer
BDBV-SS-1	216
BDBV-SS-2	149
BDBV-SS-3	72
Neg. control	<10
