# Supplementary material for: Development and characterization of a recombinant Bundibugyo virus expressing a fluorescent reporter protein
Source: Emerg Microbes Infect. 2026 Jul 24;15(1):2709847. doi: 10.1080/22221751.2026.2709847 (PMC13435268; doi:10.1080/22221751.2026.2709847)

Supp.Fig.1

Primers to clone BDBV-2012

Frag.1 (pos.1-4120)
BDBV-1-F (plasmid seq in lowercase)
atacgactcactataGGACACACAAAAAGAATGAAG
BDBV-4120-R
TTAGATTTTGAGTCCGAGTG
Frag.2 (pos.4120-8280)
BDBV-4120-F
CGGACTCAAAATCTAAGGTG
BDBV-8280-R
TTTTTCTTAATCTTCATCAAGTATC
Frag.3 (pos.8280-13430)
BDBV-8280-F
GATGAAGATTAAGAAAAAGAC
BDBV-13430-R
TAACAGTGGCATTCTCACC
Frag.3 (pos.13430-18939)
BDBV-13430-F
AGAATGCCACTGTTAGAGG
BDBV-18939-R (plasmid seq in lowercase)
atgccatgccgacccGGACACACAAAAAAGAGAAAAC

Schematic overlapping fragments

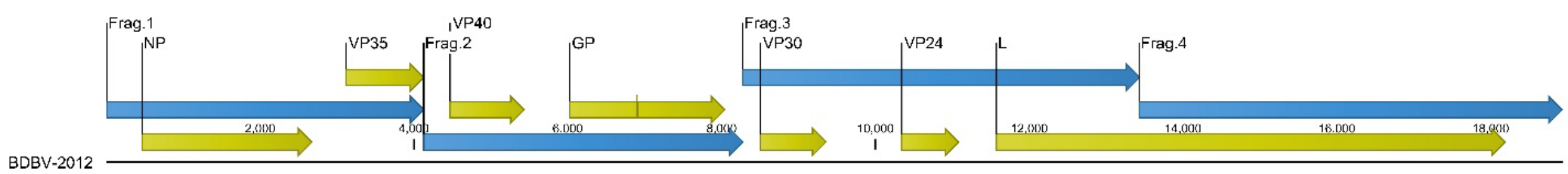

Supplement: Supplementary file 2 [file TEMI_A_2709847_SM3042.pdf]
