# Supplementary material for: Development and characterization of a recombinant Bundibugyo virus expressing a fluorescent reporter protein
Source: Emerg Microbes Infect. 2026 Jul 24;15(1):2709847. doi: 10.1080/22221751.2026.2709847 (PMC13435268; doi:10.1080/22221751.2026.2709847)
Supplement: Supplementary file 3 [file TEMI_A_2709847_SM3043.pdf]

Supp.Fig.2

A

```
                                                   20                  40                  60
BDBV UGA-2007 (NC_014373)     MVTSGILQLPRERFRKTSFFVWVIILFHKVFPIPLGVVHNNTLQVSDIDKLVCRDKLSSTSQLKSVGLN  69
BDBV DRC-2012 (KC545395)      MVTSGILQLPRERFRKTSFFVWVIILFHKVFPIPLGVVHNNTLQVSDIDKLVCRDKLSSTSQLKSVGLN  69
BDBV DRC-2026 (PP_006XHKB)    MVTSGILQLPRERFRKTSFFVWVIILFHKVFPIPLGVVHNNTLQVSDIDKLVCRDKLSSTSQLKSVGLN  69
                                         80                 100                 120
BDBV UGA-2007 (NC_014373)     LEGNGVATDVPTATKRWGFRAGVPPKVVNYEAGEWAENCYNLDIKKADGSECLPEAPEGVRGFPRCRYV 138
BDBV DRC-2012 (KC545395)      LEGNGVATDVPTATKRWGFRAGVPPKVVNYEAGEWAENCYNLDIKKADGSECLPEAPEGVRGFPRCRYV 138
BDBV DRC-2026 (PP_006XHKB)    LEGNGVATDVPTATKRWGFRAGVPPKVVNYEAGEWAENCYNLDIKKADGSECLPEAPEGVRGFPRCRYV 138
                               140                 160                 180                 200
BDBV UGA-2007 (NC_014373)     HKVSGTGPCPEGYAFHKEGAFFLYDRLASTIIYRSTTFSEGVVAFLILPETKKDFFQSPPLHEPANMTT 207
BDBV DRC-2012 (KC545395)      HKVSGTGPCPEGFAFHKEGAFFLYDRLASTIIYRSTTFSEGVVAFLILPKTKKDFFQSPPLHEPANMTT 207
BDBV DRC-2026 (PP_006XHKB)    HKVSGTGPCPEGFAFHKEGAFFLYDRLASTIIYRSTTFSEGVVAFLILPKTKKDFFQSPPLHEPANMTT 207
                                           220                 240                 260
BDBV UGA-2007 (NC_014373)     DPSSYYHTVTLNYVADNFGTNMTNFLFQVDHLTYVQLEPRFTPQFLVQLNETIYTNGRRSNTTGTLIWK 276
BDBV DRC-2012 (KC545395)      DPSSYYHTVTLNYVADNFGTNMTNFLFQVDHLTYVQLEPRFTPQFLVQLNETIYTNGRRSNTTGTLIWK 276
BDBV DRC-2026 (PP_006XHKB)    DPSSYYHTVTLNYVADNFGTNMTNFLFQVDHLTYVQLEPRFTPQFLVQLNETIYTNGRRSNTTGTLIWK 276
                                 280                 300                 320                 340
BDBV UGA-2007 (NC_014373)     VNPTVDTGVGEWAFWENKKNFTKTLSSEELSVIFVPRAQDPGSNQKTKVTPTSFANNQTSKNHEDLVPE 345
BDBV DRC-2012 (KC545395)      VNPTVDTGVGEWAFWENKKNFTKTLSSEELSVILVPRAQDPGSNQKTKVTPTSFANNQTSKNHEDLVPK 345
BDBV DRC-2026 (PP_006XHKB)    VNPTVDTGVGEWAFWENKKNFTKTLSSEELSVIFVPRAQDPGSNQKTKVTPTSFANNQTSKNHEDLVPE 345
                                             360                 380                 400
BDBV UGA-2007 (NC_014373)     DPASVVQVRDLQRENTVPTPPPDTVPTTLIPDTMEEQTTSHYEPPNISRNHQERNNTAHPETLANNPPD 414
BDBV DRC-2012 (KC545395)      DPASVVQVRDLQRENTVPTSPLNTVPTTLIPDTMEEQTTSHYELPNISGNHQERNNTAHPETLANNPPD 414
BDBV DRC-2026 (PP_006XHKB)    DPASVVQVRDLQRENTVPTSPLDTVPTTLIPDTMEEQTTSHHELPNISGNHQERNNTAHPETLANNPPD 414
                                   420                 440                 460                 480
BDBV UGA-2007 (NC_014373)     NTTPSTPPQDGERTSSHTTPSPRPVPTSTIHPTTRETHIPTTMTTSHDTDSNRPNPIDISESTEPGPLT 483
BDBV DRC-2012 (KC545395)      NTTPSTPPQDGERTSSHTTPSPRPVPTSTIHPTTRETQIPTTMITSHDTDSNRPNPIDISESTEPGLLT 483
BDBV DRC-2026 (PP_006XHKB)    NTTPSTPPQDGERTSSHTTPSPRPVPTSTIHPTTRETHIPTTMITSHDTDSNRPNPIDISESTEPGLLT 483
                                               500                 520                 540
BDBV UGA-2007 (NC_014373)     NTTRGAANLLTGSRRTRREITLRTQAKCNPNLHYWTTQDEGAAIGLAWIPYFGPAAEGIYTEGIMHNQN 552
BDBV DRC-2012 (KC545395)      NTIRGVANLLTGSRRTRREITLRTQAKCNPNLHYWTTQDEGAAIGLAWIPYFGPAAEGIYTEGIMHNQN 552
BDBV DRC-2026 (PP_006XHKB)    NTIRGVANLLTGSRRTRREITLSTQAKCNPNLHYWTTQDEGAAIGLAWIPYFGPAAEGIYTEGIMHNQN 552
                                     560                 580                 600                 620
BDBV UGA-2007 (NC_014373)     GLICGLRQLANETTQALQLFLRATTELRTFSILNRKAIDFLLQRWGGTCHILGPDCCIEPHDWTKNITD 621
BDBV DRC-2012 (KC545395)      GLICGLRQLANETTQALQLFLRATTELRTFSILNRKAIDFLLQRWGGTCHILGPDCCIEPHDWTKNITD 621
BDBV DRC-2026 (PP_006XHKB)    GLICGLRQLANETTQALQLFLRATTELRTFSILNRKAIDFLLQRWGGTCHILGPDCCIEPHDWTKNITD 621
                                                   640                 660
BDBV UGA-2007 (NC_014373)     KIDQIIHDFIDKPLPDQTDNDNWWTGWRQWVPAGIGITGVIIAVIALLCICKFLL 676
BDBV DRC-2012 (KC545395)      KIDQIIHDFIDKPLPDQTDNDNWWTGWRQWVPAGIGITGVIIAVIALLCICKFLL 676
BDBV DRC-2026 (PP_006XHKB)    KIDQIIHDFIDKPLPDQTDNDNWWTGWRQWVPAGIGITGVIIAVIALLCICKFLL 676
```

B

		1	2	3
BDBV UGA-2007 (NC_014373)	1		14	12
BDBV DRC-2012 (KC545395)	2	97.9		6
BDBV DRC-2026 (PP_006XHKB)	3	98.2	99.1	
