# Supplementary material for: Development and characterization of a recombinant Bundibugyo virus expressing a fluorescent reporter protein
Source: Emerg Microbes Infect. 2026 Jul 24;15(1):2709847. doi: 10.1080/22221751.2026.2709847 (PMC13435268; doi:10.1080/22221751.2026.2709847)
Supplement: Supplementary file 4 [file TEMI_A_2709847_SM3040.docx]

**Materials and Methods**

**Cell culture and biosafety**

Human hepatoma (Huh7) (Apath, LLC) and Vero CCL-81 cells (ATCC) were propagated in Dulbecco’s modified Eagle medium (DMEM; Gibco) supplemented with 1 × non-essential amino acids, 5% (v/v) fetal bovine serum, 100 units/mL penicillin, and 100 μg/mL streptomycin (Gibco). All cells were incubated at 37 °C in 5% CO_2._ All work with infectious viruses was conducted in a BSL-4 facility at the Centers for Disease Control and Prevention (CDC, USA) following established BSL-4 standard operating procedures. All recombinant virus work was approved by the Institutional Biosafety Committee (IBC).

**Plasmid Construction and rescue of recombinant virus having fluorescent reporter**

Recombinant BDBV was constructed as previously described [1,2]. Briefly, viral RNA from an acute case EboBund-122 2012 (GenBank accession KC545395.1) was used as a template to amplify 4 overlapping fragments to assemble a full-length clone in the T7 transcription vector (supp fig 1). These fragments were gel-purified and used to assemble a full-length clone into the T7 transcription vector. The final plasmid contained the full-length anti-genome (viral complementary sense) preceded by the T7 RNA polymerase promoter and followed by the hepatitis delta virus ribozyme and T7 polymerase terminator. The ZsG open reading frame and porcine teschovirus 2A self-cleaving peptide were fused at the amino terminus of NP. Rescue of recombinant virus was performed in Huh7 cells as previously described ([1,2]). Briefly, a ~70% confluent monolayer of Huh7 cells grown in a 12-well plate was transfected with 0.5 μg pBDBV-ZsG, 0.5 μg pC-L, 0.25 μg pC-NP, 0.05 μg pC-VP35, 0.05 μg pC-VP30, and 0.5 μg of codon-optimized pC-T7. Supernatants were harvested 3 days after transfection and blind-passaged twice in Huh7 cells. Viral stocks were sequenced by NGS on the MiniSeq platform (Illumina, San Diego, CA, USA). The viral genomic sequences were identical to those in the full-length plasmids.

**Immunofluorescence antibody staining**

For immunofluorescence assays (IFA), Vero-CCL-81 cells were seeded onto 96-well plates (Costar) and infected with the recombinant viruses at MOI of 0.1. Three days post infection, cells were fixed with 10% formalin for 30 min followed by permeabilization with 0.1% Triton X-100 in phosphate-buffered saline (PBS) for 15 min and blocking with 1% bovine serum albumin for 1 h. A polyclonal rabbit anti-EBOV antibody (generated by injecting rabbits with inactivated EBOV; Viral Special Pathogens Branch, CDC) was used to stain the cells for 1 hour at room temperature followed by 2 washes with PBS and treatment with secondary anti-rabbit 594 antibody. Cell nuclei were stained with NucBlue (Thermo Fisher). Cells were observed and imaged using an EVOS digital inverted microscope (Thermo Fisher) with 20 X objective.

**Virus titer determination and growth curves**

The titer of BDBV-WT was determined by standard 50 % tissue culture infectious dose (TCID_50_) protocol using the Reed and Muench method [3] on Vero-CCL-81 and Huh7 cells, utilizing an IFA. Briefly, cells were inoculated with 10-fold serial dilutions of the virus. At 3 days post infection (dpi), cells were fixed and IFA was performed on them as mentioned above. The titration was performed similarly on rBDBV-ZsG, using reporter-driven ZsGreen fluorescence to score positive wells.

For growth kinetics, the Huh7 cells were seeded at 50 % confluency in 6-well plate (Costar) one day prior to infection. The cells were infected with different viruses at MOI of 0.05. After 1 hour of absorption at 37°C, the inoculum was removed, cells were washed with 1X PBS, and DMEM medium with 5 % serum was added. The cell culture supernatants were collected at 0, 1-, 2-, 3- and 4- dpi. The virus was quantified by TCID_50_ as mentioned above.

**Testing of antiviral compounds**

For antiviral assays, Huh7 cells were seeded 1 X 10^4^ cells/well of a 96-well plate in Fluorobrite cell culture media supplemented with 10% FBS, NEAA, 1% glutamax, and penicillin/streptomycin 1 day prior to the assay. All antiviral compounds were obtained from MedChemExpress. Antiviral properties of different compounds were screened using rBDBV-ZsG and wild type BDBV 2012 strain (BDBV-WT) as mentioned [1,4,5]. Cell viability was determined for the antiviral compounds using Cell-Titer Glo (Promega). All the antiviral testing experiments were done in quadruplicates and values were normalized to mock transfected and DMSO only treated cells. The normalized values were used to fit a 4-parameter equation to semi-log plots of the concentration-response data using GraphPad Prism v10.4.0 (GraphPad Software). The 50% effective concentration (EC_50_) of each antiviral compound was calculated by interpolating the normalized values. The 50% cytotoxic concentration of each compound (CC_50_) was calculated similarly. The selectivity index (SI) was calculated by dividing CC_50_ by EC_50_. Z prime (Z’) score was calculated to determine the suitability for antiviral screening. Z’ was calculated as Z’ =1-(3 X (σP +σN)/|μp-μN|, where σP is standard deviation of positive control, σN is standard deviation of negative control, μp is mean of positive control, and μN is mean of negative control. Z’ values between 0.5 and 1.0 were considered acceptable [6].

**Patient Serum Sample Selection**

CDC archived samples are residual clinical samples that were approved for use in this research by the Centers for Disease Control and Prevention Institutional Review Board (IRB Protocol #7276).

**Neutralization assays**

Neutralization assays were performed on archived clinical sera and pan-ebolavirus antibody ADI-15878 as previously described [7]. The antibody ADI-15878 was made in-house using the sequence mentioned previously (GenBank accession KU602365, KU602366). 10,000 cells/well were seeded onto 96-well plates one day prior to the experiment. Serial 5-fold dilutions of antibodies or human serum samples were mixed with 200 TCID_50_ of rBDBV-ZsG. After the virus-antibody/sample mixture was incubated for 1 h at 37^◦^C, it was added to Vero-CCl-81 cell monolayers in triplicate. Cells were incubated for 72 h at 37^◦^C and the fluorescence signal was measured using a Cytation plate reader (BioTek).

All experiments were done in triplicate. Data were analyzed using GraphPad Prism v10.4.0 (GraphPad Software). Fluorescence values of ZsG expression were normalized to fluorescence in non-infected cells. The resulting normalized values were used to fit a 4-parameter equation to semi-log plots of the concentration-response data. Dilution of the antibodies/samples inhibiting 50% of ZsG expression (IC_50_) was calculated by interpolating the values.

References

[1]	McMullan LK, Flint M, Chakrabarti A, et al. Characterisation of infectious Ebola virus from the ongoing outbreak to guide response activities in the Democratic Republic of the Congo: a phylogenetic and in vitro analysis. Lancet Infect Dis. 2019;19(9):1023-1032.

[2]	Albarino CG, Wiggleton Guerrero L, Lo MK, et al. Development of a reverse genetics system to generate a recombinant Ebola virus Makona expressing a green fluorescent protein. Virology. 2015;484:259-264.

[3]	Reed LM, H. A simple method for estimating fifty percent endpoints. Am J Epidemiol. 1938;27(3):493-497.

[4]	Jain S, Shrivastava-Ranjan P, Montgomery JM, et al. Characterization of minigenome systems for Hantaan and Seoul hantaviruses. Virus Res. 2026;363:199678.

[5]	Jain S, Shrivastava-Ranjan P, Flint M, et al. Development of reverse genetic tools to study Chapare and Machupo viruses. Virology. 2023;588:109888.

[6]	Zhang J-H, Chung DYT, Oldenburf K. A simple statistical parameter for use in evaluation and validation of high throughput screening assays. Journal of Biomolecular Screening. 1999;4.

[7]	Jain S, Lo MK, Kainulainen MH, et al. Development of a neutralization assay using a vesicular stomatitis virus expressing Nipah virus glycoprotein and a fluorescent protein. Virology. 2023;587:109858.

**Supplementary Figure Legend:**

**Supp Fig 1:**

Primers to generate the overlapping fragments for rBDBV cDNA clone. Schematic shows the overlapping fragments.

**Supp Fig 2:**

A) Alignment of BDBV 2007, 2012 and 2026 glycoprotein amino acid sequences

B) **Pairwise comparison of BDBV glycoprotein amino acid sequences**: The matrix shows the relationship between the glycoprotein precursors of BDBV 2007, 2012 and 2026 strains. For each pair, the upper value indicates the total number of amino acid differences, and the lower value represents the percentage of sequence identity.

**Supp Fig 3:**

A) 50% cell cytotoxicity (CC_50_), % effective inhibition concentration (EC_50_), and selective index (SI) values for each compound against BDBV-2012-WT and rBDBV-2012-ZsG.
B) 50 % effective inhibition concentration (IC_50_) and neutralization titer values of each indicated antibody and serum samples against rBDBV-ZsG.
